# The influence of maternal representations of the child on parenting behaviors: A longitudinal study from pregnancy to 4 years postpartum

**DOI:** 10.1002/imhj.70122

**Published:** 2026-10-09

**Authors:** Joohee Lee, Matthew J. Marvin, Alexandra L. Ballinger, G. Anne Bogat, Amy K. Nuttall, Alytia A. Levendosky

**Affiliations:** ^1^ Department of Psychology Michigan State University East Lansing Michigan USA; ^2^ Department of Human Development and Family Studies Michigan State University East Lansing Michigan USA

**Keywords:** intimate partner violence (IPV), maternal representations, mother–child relationships, parenting behaviors, pregnancy, working models

## Abstract

Maternal representations of the child begin forming during pregnancy and evolve through interactions with the baby. Research suggests that prenatal representations may have a more stable impact on parenting than postnatal representations, but their long‐term impact remains unclear. This study examines (1) how prenatal and 1‐year postnatal representations are associated with parenting when child is age 4 and (2) whether prenatal representations have a more enduring influence on later caregiving than postnatal representations by exploring how stability or shifts in representations across time shape parenting. One hundred fifty‐five US mothers, oversampled for intimate partner violence and low income, participated in this longitudinal study. Prenatal balanced representations were significantly associated with more sensitive and less unresponsive parenting, whereas disengaged representations were linked to lower sensitivity and greater unresponsiveness. Postnatal representations predicted sensitive parenting but were not significantly associated with intrusive or unresponsive behaviors. Examination of changes in prenatal to postnatal representations revealed that mothers who shifted from balanced (prenatal) to non‐balanced (postnatal) representations displayed higher sensitivity than those who remained non‐balanced. Results highlight the consistent impact of prenatal representations on parenting, emphasizing the need for timely interventions during pregnancy and the early postpartum period to promote positive parenting.

## 1 INTRODUCTION

Parenting shapes children's emotional, behavioral, and social outcomes from infancy to early childhood and beyond (Cooke et al., 2022; Morris et al., 2017; Valcan et al., 2018; Zimmer‐Gembeck et al., 2022). Among the factors influencing parenting, maternal representations (i.e., mothers’ internal working models of their child and relationship with their child) play a critical role in guiding caregiving behaviors (Benoit et al., 1997; Dayton et al., 2010; Lindstedt et al., 2024). These representations, developed during pregnancy and refined postnatally, form the foundation for maternal interactions with their children (Benoit et al., 1997; Dayton et al., 2010; Stern, 1995). These representations are also influenced by maternal experiences in close relationships, including mothers’ own experiences of being parented (Huth‐Bocks, Levendosky, Theran, et al., 2004) and their experiences in their adult romantic relationships (Huth‐Bocks, Levendosky, Bogat, et al., 2004). Childhood abuse and intimate partner violence (IPV) negatively affect these representations (Huth‐Bocks, Levendosky, Bogat, et al., 2004; Kobayashi et al., 2021). While extant research has documented that these maternal representations affect maternal parenting at age 1 year (Dayton et al., 2010; Theran et al., 2005), the stability and impact of their effects on parenting over time, particularly as children transition into preschool age, remain underexplored. This present study investigates whether maternal representations during pregnancy, as well as one‐year postpartum representations, influence parenting behaviors when children are 4 years old in a group of low‐income women oversampled for IPV.

During pregnancy, as a woman prepares psychologically for a new caregiving role (Cohen & Slade, 2000), she develops maternal representations of her unborn baby, that reflect her thoughts, emotions, and expectations regarding the child and her future caregiving role (Zeanah et al., 1990). These early prenatal representations become part of a broader maternal caregiving behavioral system, a biologically driven system that motivates mothers to provide care, protection, and emotional support to their children, paralleling the child's instinct to seek comfort and security from their caregivers (Aber et al., 1999; George, 1999).

Similar to attachment representations, maternal representations can be classified using codes based on narrative responses to an interview, such as the Working Model of the Child Interview (WMCI: Zeanah et al., 1996). These categories include balanced or one of two non‐balanced categories: disengaged and distorted (Zeanah et al., 1996). Balanced representations are characterized by emotional warmth and acceptance, demonstrating a developed capacity for child‐focused reflective functioning, that is, the ability to identify internal states as underlying the observed behavior of the child (Zeanah et al., 1996). The interviews of those classified as disengaged are typically lacking in detail and characterized by a sense of aloofness or indifference toward the child (Zeanah et al., 1996). Finally, the distorted classification is characterized by emotion dysregulation and an anxious orientation toward the child and caregiving (Zeanah et al., 1996).

**Key Points**

- Balanced prenatal representations were associated with higher sensitivity and lower unresponsiveness in parenting at age 4, whereas disengaged prenatal representations were linked to lower sensitivity and greater unresponsiveness.
- Balanced postnatal maternal representations were associated with greater maternal sensitivity compared with disengaged postnatal representations.
- While parenting behaviors of mothers who remained balanced throughout pregnancy and one‐year postpartum did not significantly differ from those who shifted from non‐balanced to balanced, mothers who shifted from balanced to non‐balanced displayed greater sensitivity than those who remained non‐balanced across both periods.

**Statement of relevance to infant and early childhood mental health**

Maternal representations of children shape parenting behaviors, further influencing overall child development. This study demonstrates an association between early‐established maternal representations during pregnancy and parenting behaviors at age 4. These findings provide a foundation for future research on the long‐term influence of prenatal maternal representations beyond early childhood and underscore the importance of identifying and fostering adaptive maternal representations at the early stages of their formation to support positive parenting outcomes.

These categories provide a critical framework for understanding how maternal representations guide caregiving behaviors (Dayton et al., 2010; Edwards et al., 2021; Sokolowski et al., 2007). Balanced representations facilitate an adaptive and flexible understanding of the child, allowing mothers to provide sensitive care that is predicated on an accurate understanding of their child's needs. On the other hand, mothers with disengaged representations may struggle to emotionally connect with their children, demonstrating difficulty in recognizing and responding appropriately to their children's emotional cues. Lastly, mothers with distorted representations may impose their own needs or anxieties onto the child, increasing the likelihood of engaging in more intrusive and controlling parenting.

Importantly, maternal representations are affected by external stressors, and thus how mothers perceive themselves, their role as mothers, and their connection with their unborn and newborn children (Huth‐Bocks, Levendosky, Bogat, et al., 2004; Huth‐Bocks et al., 2011; Slade et al., 1999). IPV, a traumatic stressor perpetrated by a romantic partner, may damage mothers’ attachment system, increase parenting stress, and reduce their emotional capacity to adequately attend to their children's needs, which in turn negatively affects parenting practices and the quality of mother‐child attachment and relationships (Huth‐Bocks & Hughes, 2008; Huth‐Bocks, Levendosky, Theran, et al., 2004; Kobayashi et al., 2021; Levendosky et al., 2006; Noonan & Pilkington, 2020). These findings underscore the need to address psychosocial risk factors, such as IPV, in understanding the association between maternal representations and caregiving behaviors.

Prenatal representations (i.e., those developed during pregnancy) are consistently linked to parenting during infancy. Ammaniti (1991) found that prenatal maternal representations predicted parenting behavior at 1 year of age. Using the same sample as the present study, Dayton et al. (2010) found that prenatal maternal representations were associated with parenting behaviors when the child was 1 year old. Specifically, balanced representations predicted more positive parenting, while disengaged representations and distorted representations were associated with more controlling and hostile behaviors, respectively. Additionally, one recent meta‐analysis (Foley & Hughes, 2018) drawing from 14 studies revealed that a mother's thoughts and feelings regarding her unborn child, such as her expectations, anxieties, and emotional bonds, were modestly but consistently related to the quality of her parenting behaviors following birth. Mothers with more positive and secure prenatal representations of their children showed more sensitive, responsive, and nurturing behavior when interacting with their infants (Foley & Hughes, 2018).

Postnatal working models (i.e., those assessed after birth) also play a critical role in shaping parenting behaviors. For example, a systematic review by Vreeswijk et al. (2012) synthesized results from 24 studies using the WMCI and concluded that balanced maternal representations are robustly linked to sensitive, responsive parenting, while disengaged and distorted representations are associated with less optimal caregiving. Furthermore, Korja et al. (2010) found that postnatal maternal representations were related to the quality of mother‐infant interactions among 1‐year‐old children. Sokolowski et al. (2007) investigated mothers of 17‐ to 20‐month‐old infants, finding that mothers with disengaged representations were less sensitive, more passive, and provided less support and guidance to their children. In a cross‐sectional study examining the association between mothers’ representations of their preschool‐aged children and various parenting behaviors, mothers with balanced representations were more sensitive, less negative, and provided greater support for their children's learning compared to mothers with non‐balanced representations (Edwards et al., 2021). Overall, research on the associations between both pre‐ and postnatal representations and later parenting behavior aligns with theoretically informed predictions, suggesting that balanced representations facilitate sensitive caregiving, while disengaged and distorted representations are associated with negative parenting behaviors. However, there is a lack of research examining whether early maternal representations continue to shape caregiving as children progress through early childhood.

Prenatal maternal representations are generally stable but can also change in response to personal experiences, external stressors, and the increasing complexity of child development. Shifts in maternal representations are important for understanding variations in caregiving behaviors. For example, using the same sample as the present study, Theran et al. (2005) examined stability and change in these representations from pregnancy to when children were age 1 and how change influenced maternal parenting. Mothers who maintained balanced representations from pregnancy to 1 year postpartum showed higher quality caregiving behaviors (higher sensitivity/warmth, lower disengagement) when their children were 1 year old compared with those who shifted to balanced representations from non‐balanced ones. Conversely, mothers who shifted to non‐balanced representations from balanced ones provided higher quality caregiving than those who remained non‐balanced. These results suggest that prenatal maternal representations may have a more enduring effect on parenting behaviors than postnatal representations, highlighting the dynamic nature of maternal representations and their implications for caregiving.

Despite substantial research on maternal representations and caregiving, several gaps remain in understanding maternal representations and their impact on caregiving. Most prior work has focused on how prenatal or early postnatal maternal representations predict parenting during infancy or toddlerhood, but little is known about whether these early representations continue to shape parenting as children reach the preschool years, a time marked by increasing autonomy and new developmental challenges (Alwaely et al., 2021; Conti‐Ramsden & Durkin, 2012; Erikson, 1963; Gomes & Pereira, 2014). A unique contribution of this study is the extension of observed parenting behavior when children are 4 years of age, allowing us to test whether prenatal and early postnatal maternal representations have a lasting impact as children mature. In addition, we directly examine whether stability or change in mothers’ working models of their child from pregnancy to infancy—such as remaining balanced, shifting to balanced, or shifting away from balanced—relates to later parenting quality. Addressing these questions clarifies whether prenatal and/or postnatal representations exert enduring effects or show differential associations with later parenting and helps identify critical windows for intervention to support at‐risk mothers and children as they transition through early developmental periods.

### 1.1 The present study

Drawing on prior findings that maternal representations—categorized as balanced, disengaged, or distorted—predict parenting behaviors during infancy (Dayton et al., 2010), our first research question investigated whether prenatal and one‐year postnatal maternal representations were differentially associated with parenting behaviors when children were 4 years old. For prenatal representations, we hypothesized that there would be group differences in parenting based on representation type, such that (a) mothers with balanced prenatal representations would display significantly higher sensitivity than mothers with disengaged or distorted representations; (b) mothers with disengaged prenatal representations would display significantly higher unresponsiveness than mothers with balanced or distorted representations; and (c) mothers with distorted prenatal representations would display significantly higher intrusive/controlling parenting behaviors than mothers with balanced or disengaged representations. We hypothesized the same pattern of group differences for postnatal representations assessed when children were 1 year old. Specifically, we expected that (a) mothers with balanced postnatal representations would display higher sensitivity; (b) mothers with disengaged postnatal representations would display higher unresponsiveness; and (c) mothers with distorted postnatal representations would display higher intrusive/controlling parenting behaviors, relative to the other postnatal representation groups.

The second research question examined how stability or change in maternal working model classifications from pregnancy to 1 year postpartum influenced caregiving behaviors when the child was 4 years old. For this question, maternal representations were categorized into two groups at each time point: balanced and non‐balanced (cf. Theran et al., 2005). Specifically, we compared mothers who remained balanced with those who shifted from non‐balanced to balanced, as well as mothers who remained non‐balanced with those who shifted from balanced to non‐balanced. These comparisons allowed us to test whether early maternal representations exerted a lasting and potentially stronger influence on caregiving behaviors by examining how differences in prenatal representations affected outcomes when groups shared the same postnatal representations. The hypotheses were (a) mothers who remained balanced would show better parenting behaviors than those who shifted from non‐balanced to balanced representations, and (b) mothers who shifted from balanced to non‐balanced representations would show better parenting behaviors than those who remained non‐balanced. Additionally, because parenting is influenced by contextual factors—including stressful life events, socioeconomic stressors, and child characteristics—we included IPV measured during pregnancy and at child age 4, prenatal monthly household income, and child internalizing and externalizing symptoms at age 4 as covariates in both research questions.

## 2 METHOD

### 2.1 Participants

The current study used a subset of data collected between 2000 and 2004 from a larger, longitudinal study examining risk and protective factors associated with exposure to prenatal IPV. Pregnant women were primarily recruited through flyers posted across various local agencies and clinics in a medium‐sized city in the Midwest. Participants were screened for inclusion based on four criteria: (1) aged between 18 and 40 years, (2) proficient in English to complete interviews and surveys, (3) had been in a romantic relationship for at least six weeks during pregnancy, and (4) in their last trimester at the time of the initial interview. Additionally, participants were oversampled for exposure to IPV such that half of the participants reported experiencing IPV during pregnancy. Participants were diverse with respect to race and ethnicity, education, and marital status. See Table 1 for participant demographics. The present study used data from pregnancy to when children were 4 years old, with data collection waves occurring during the third trimester of pregnancy (Time 1; T1), when the child was 1year old (Time 2; T2), and when the child was 4 years old (Time 3; T3). The full longitudinal sample included 206 participants assessed at T1, T2, and T3. Analytic sample sizes varied depending on data availability for maternal representations and parenting outcomes. Analyses examining prenatal representations (T1) predicting parenting at T3 included 155 participants with complete representation and parenting data. Analyses examining postnatal representations (T2) predicting parenting at T3 included 154 participants. Finally, analyses examining change in representations from T1 to T2 predicting parenting at T3 included 150 participants. The only demographic difference between participants who dropped from the study and those who were retained was that participants who dropped out reported significantly lower monthly incomes at T1 than participants who were retained *t*(203) = −1.73, *p* = .04. WMCI classifications at T1 and T2 were not significantly different in the group of participants who were dropped from analyses and those who were retained.

**TABLE 1:** Maternal demographic characteristics.

Demographic variable	*Mean*	*SD*
Age (years)	25.3	4.91
Monthly household income (prenatal)	1929.55	1495.00
	Frequency	Percent
Marital status		
Single, never married	71	47.3
Married	66	44
Separated	4	2.7
Divorced	8	5.3
Widowed	1	0.7
Currently living with partner		
Yes	102	68
No	48	32
Race and ethnicity		
Native American	2	1.3
Asian American/Pacific Islander	2	1.3
Black, African American	37	24.7
Latina, Hispanic, Chicano	8	5.3
Biracial (mixed)	8	5.3
Caucasian, White	93	62
Education		
GED/High school diploma	66	44
Some college	52	34.7
AA degree	7	4.7
BA/BS degree	12	8
Graduate degree	8	5.3
Other	5	3.3
Work outside the home		
Yes	65	43.3
No	85	56.7

### 2.2 Measures

#### 2.2.1 Maternal representation of the infant

The Working Model of the Child Interview (WMCI; Zeanah et al., 1994) assessed pre‐ and postnatal maternal representations at T1 and T2, respectively. The WMCI is a semi‐structured interview that assesses parental internal perceptions and subjective experiences of the child's personality and the parent–child relationship. The prenatal WMCI was adapted for the third trimester of pregnancy by changing the interview questions to future tense, which has been a reliable and valid method for assessing prenatal representations (Benoit et al., 1997). The WMCI at both T1 and T2 took about one hour to complete. Interviews were audiotaped and later transcribed verbatim for coding. The coding scheme developed by Zeanah et al. (1986) was used to categorize representations into three groups: one balanced and two non‐balanced groups—disengaged and distorted (Zeanah et al., 1996). Trained graduate students in clinical psychology coded the transcripts. Inter‐rater reliability was assessed using percent agreement and Cohen's kappa for the overall classifications. At T1, the percent agreement for overall classification was 96%, with a kappa of  .94 (*p* = .001). At T2, percent agreement averaged 80%, with an average kappa of  .62 (*p* = .001). Disagreements were resolved through discussion and consensus coding.

#### 2.2.2 Maternal parenting

Maternal parenting behavior was coded from a 14‐min mother–child interaction task administered at T3, using the revised version of the Dyadic Parent–Child Interaction Coding System (DPICS) (Robinson & Eyberg, 1981). This version was adapted by the MSU Family Project (Whipple et al., 1993), based on Belsky's Parent–Child Interaction Coding System (Belsky et al., 1991), and incorporated elements from the Emotional Availability Scales (Biringen, 2000). The task consisted of a 7‐min child‐directed portion, where the child engaged in free play, and a 7‐min parent‐directed portion, where the parent directed the play activity. At the end of the task, mothers were instructed to have their children clean up the play area. All interactions were video recorded and later coded by trained raters. The codes used in the current study were sensitivity, intrusive/overcontrolling, and unresponsive/unavailable/undercontrolling. Sensitive parenting was defined as the ability to accurately perceive and promptly and appropriately respond to a child's signals, showing empathy and providing a supportive presence that enhances the child's sense of security, comfort, and development. Intrusive and overcontrolling parenting was defined as excessively controlling behavior that disrupts the child's activities and lacks empathy, driven by a parental agenda. Unresponsive, unavailable, and undercontrolling parenting was defined as a parent's failure to provide necessary control, support, or involvement, characterized by inaction or token gestures.

Maternal parenting behaviors were coded at 1‐minute intervals using a 5‐point Likert‐type scale based on qualitative and quantitative aspects of the interaction, including tone, timing, contingency, and emotional attunement. For each parenting behavior, scores were averaged across the seven 1‐min segments within each play context (child‐directed and mother‐directed play). Each point on the scale was anchored by clearly defined behavioral descriptors in the coding manual to ensure reliability and consistency. The final composite score for each parenting behavior was then calculated by averaging the scores from both play contexts. Higher scores indicated a greater presence of the targeted behavior. This scoring approach, previously employed in published research by Kobayashi et al. (2021), reflects the intent to capture consistent parenting patterns across different interactional demands. Reliability data were collected on 20% of observations, and interrater reliability, assessed using weighted kappa coefficients, ranged from 0.84 to 0.94 across the codes.

#### 2.2.3 Intimate partner violence

Prenatal IPV was assessed using the Severity of Violence Against Women Scales (SVAWS; Marshall, 1992) at the pregnancy interview. The SVAWS is a 46‐item questionnaire assessing violent behaviors and threats a woman has experienced from her partner. Participants were asked to report on violence they had experienced with their current partner during this pregnancy. Each item is rated on a 4‐point scale ranging from Never to Many Times. Items include psychological threats, physical, and sexual abuse. In this sample, internal consistency was high for all subscales (*α* = 0.91–1.00). A total sum of all items was used to reflect IPV during the current pregnancy.

#### 2.2.4 Children's internalizing and externalizing problems

The Child Behavior Checklist (CBCL; Achenbach, 1991) assessed children's emotional and behavioral problems. Mothers rated 112 items describing child behaviors over the past 6 months on a 3‐point scale ranging from 0 (Not True) to 2 (Very True or Often True). The measure yields broadband Internalizing and Externalizing scales. In the present sample, internal consistency was good for the Externalizing scale (*α* = 0.85) and adequate for the Internalizing scale (*α* = 0.69).

#### 2.2.5 Demographics

Participant demographics, including monthly income, race and ethnicity, marital status, and educational attainment, were assessed during the pregnancy interview.

### 2.3 Procedures

After the preliminary screening assessment, women were scheduled for an in‐person interview during their third trimester of pregnancy. This lab visit included the semi‐structured interview of maternal prenatal representations. When children were 1 year old, the participants and their children returned to the lab for another visit, during which maternal postnatal representations were assessed through another semi‐structured interview. At the 4‐year follow‐up, mother‐child dyads returned to the project offices where they were videorecorded participating in a parent‐child interaction task that was later coded for maternal parenting behaviors. The study was approved by the institutional IRB. Participants completed informed consent and were compensated for their participation at each study visit.

### 2.4 Data analysis plan

Generalized linear models (GLMs) were used instead of multivariate analysis of variance (MANOVA) because Levene's test indicated a violation of the homogeneity of variance assumption for unresponsiveness, *F*(2, 152) = 3.62, *p* = .029. GLMs are less sensitive to violations of this assumption (Salinas Ruíz et al., 2023).

For the first research question, we conducted two sets of GLMs: one set tested the association between prenatal maternal representation type (balanced, disengaged, or distorted) and parenting behaviors, and the other set tested the association between postnatal representation type (balanced, disengaged, or distorted) and parenting behaviors. Within each time point (prenatal and postnatal), we ran three separate GLMs, one for each parenting behavior (sensitivity, intrusiveness, and unresponsiveness).

For the second research question, we conducted two additional sets of GLMs to examine differences in parenting behaviors based on the stability or change in maternal representation type between the prenatal and postnatal periods. Specifically, we compared (a) mothers who remained balanced with those who became balanced, and (b) mothers who remained non‐balanced with those who became non‐balanced. For each comparison, three separate GLMs were estimated, with the parenting behaviors of sensitivity, intrusiveness, and unresponsiveness modeled as separate outcomes.

All models included relevant covariates: prenatal household income, prenatal IPV, IPV at age 4, and child internalizing and externalizing symptoms at age 4. This analytic approach allowed us to independently assess the effects of representation type at each time point, as well as the effects of stability or change in representations over time, on later caregiving practices.

Parenting outcomes at age 4 were unavailable for 46 participants (22.3%), primarily due to attrition or incomplete observational data at that wave. Because parenting variables served as the dependent variables in all primary analyses, multiple imputation of outcomes was not implemented. Consistent with methodological recommendations cautioning against imputing dependent variables without strong auxiliary predictors (von Hippel, 2007), analyses were conducted using complete‐case samples.

## 3 RESULTS

### 3.1 Preliminary analyses

The distribution of prenatal maternal representations was 48% balanced (*n* = 75) and 52% non‐balanced (*n* = 80). 30% of the sample was disengaged (*n* = 47), and 22% were distorted (*n* = 33). The distribution of maternal representations 1 year postpartum was 60% balanced (*n* = 93) and 40% non‐balanced (*n* = 61). 19% of the sample was disengaged (*n* = 30), and 20% were distorted (*n* = 31). Of the 73 mothers classified as balanced during pregnancy, 81% (*n* = 59) remained balanced after birth, while 19% (*n* = 14) shifted to non‐balanced. Of the 77 mothers classified as non‐balanced during pregnancy, 60% (*n* = 46) remained non‐balanced, while 40% (*n* = 31) shifted to balanced.

Descriptive statistics and bivariate correlations among the study variables are presented in Table 2. Significant correlations were observed between several variables. Prenatal IPV was significantly negatively correlated with prenatal monthly household income and sensitive parenting, and positively correlated with unresponsive parenting. Age 4 IPV was also positively correlated with unresponsive parenting and negatively correlated with sensitive parenting. Prenatal IPV and age 4 IPV were significantly positively correlated. Prenatal household income was significantly positively correlated with sensitive parenting and significantly negatively correlated with intrusive parenting, unresponsive parenting, and child externalizing symptoms. Sensitive parenting was significantly negatively correlated with both intrusive parenting and unresponsive parenting. Regarding child outcomes, child internalizing symptoms at age 4 were significantly positively correlated with unresponsive parenting. Child externalizing symptoms were significantly negatively correlated with sensitive parenting and significantly positively correlated with child internalizing symptoms. No other significant associations were observed between child behavior variables and parenting or IPV variables.

**TABLE 2:** Descriptive statistics and correlations for study variables.

Variable	*M*	*SD*	1	2	3	4	5	6	7	8
1. Prenatal IPV	6.37	11.99	–							
2. Age 4 IPV	2.55	7.35	.198^**^	–						
3. Prenatal household income	1820.00	1507.29	−.230^**^	−.028	–					
4. Sensitive parenting	3.34	0.62	−.370^**^	−.236^**^	.266^**^	–				
5. Intrusive parenting	2.16	0.69	.148	.039	−.200^*^	−.415^**^	–			
6. Unresponsive parenting	2.17	0.79	.294^**^	.245^**^	−.167^*^	−.713^**^	−.106	–		
7. Child internalizing	2.09	2.38	.018	−.062	−.104	−.120	−.018	.163^*^	–	
8. Child externalizing	8.16	5.95	.128	−.013	−.158^*^	−.204^**^	.088	.104	.612^**^	–

Abbreviation: IPV, Intimate Partner Violence.

^*^ *p* < .05 and ^**^ *p* < .01.

### 3.2 Prenatal representations and parenting behaviors

#### 3.2.1 Sensitive parenting

The overall model was significant for sensitive parenting, *χ* ^2^(7) = 49.91, *p* < .001. The effect of maternal prenatal representation type on sensitive parenting was also significant, Wald *χ* ^2^(2) = 11.69, *p* = .003. As presented in Table 3, pairwise comparisons found that mothers with balanced prenatal working models exhibited higher sensitive parenting (*M *= 3.46, *SE* = 0.06) compared to those with disengaged prenatal working models (*M* = 3.10, *SE* = 0.08, *p* = .002). No significant differences were found between balanced and distorted representations (*M* = 3.36, *SE* = 0.11, *p* = 1.00) or between disengaged and distorted representations (*p* = .138).

**TABLE 3:** Comparisons of prenatal working models on parenting behaviors at 4 years postpartum.

Dependent variable	Comparison	Mean difference	*S.E*.	*p*	95% CI	
					*LL*	*UL*
Sensitivity	Balanced vs. disengaged	0.36*	0.11	.002	0.11	0.62
	Balanced vs. distorted	0.10	0.13	1.00	−0.20	0.40
	Disengaged vs. distorted	−0.26	0.13	.138	−0.58	0.05
Intrusiveness	Balanced vs. disengaged	−0.12	0.13	1.00	−0.43	0.20
	Balanced vs. distorted	−0.07	0.16	1.00	−0.45	0.31
	Disengaged vs. distorted	0.04	0.15	1.00	−0.31	0.40
Unresponsiveness	Balanced vs. disengaged	−0.41^*^	0.15	.018	−0.77	−0.05
	Balanced vs. distorted	−0.10	0.15	1.00	−0.46	0.27
	Disengaged vs. distorted	0.31	0.17	.214	−0.10	0.73

Abbreviations: CI, confidence interval; LL, lower limit; UL, upper limit.

^*^ *p* < .05.

Among the covariates, higher prenatal IPV (*B* = −.011, *SE* = .004, *p *= .006) and age 4 IPV (*B* = −.012, *SE* = .004, *p* = .005) significantly predicted lower sensitivity. Prenatal income was not significantly associated with sensitivity, and age 4 child internalizing and externalizing symptoms were not significant predictors of sensitivity.

#### 3.2.2 Intrusive parenting

For intrusive parenting, the overall model was not significant, *χ* ^2^(7) = 9.43, *p* = .223. Therefore, no further interpretation of group or covariate effects is presented.

#### 3.2.3 Unresponsive parenting

The overall model was significant for unresponsive parenting, *χ^2^* (7) = 32.05, *p* < .001.

Maternal prenatal representations significantly predicted unresponsive parenting, Wald *χ^2^* (2) = 7.69, *p* = .021. Pairwise comparisons indicated that mothers with balanced prenatal representations had lower levels of unresponsive parenting (*M* = 2.04, *SE* = 0.08) compared with those with disengaged representations (*M* = 2.45, *SE* = 0.12, *p *= .018). However, no significant differences were found between balanced and distorted representations (*M* = 2.13, *SE* = 0.13, *p* = 1.00) or between disengaged and distorted representations (*p* = .214).

Among the covariates, higher levels of prenatal IPV (*B* = .012, *SE* = .005, *p* = .016) and age 4 IPV (*B* = .019, *SE* = .009, *p* = .045) were significantly associated with greater unresponsiveness, while prenatal income and age 4 child internalizing and externalizing symptoms were not significant.

### 3.3 Postnatal representations and parenting behaviors

#### 3.3.1 Sensitive parenting

The overall model was significant for sensitivity, *χ^2^* (7) = 51.02, *p* < .001. Postnatal working model was a significant predictor of sensitive parenting, Wald *χ* ^2^(2) = 6.88, *p* = .032. Pairwise comparisons found that mothers with balanced postnatal working models exhibited higher sensitive parenting (*M* = 3.43, *SE* = 0.05) compared to those with disengaged postnatal working models (*M* = 3.11, *SE* = 0.11, *p* = .031). No significant differences were found between balanced and distorted representations (*M* = 3.29, *SE* = 0.11, *p* = .702) or between disengaged and distorted representations (*p* = .638).

For the covariates, higher levels of prenatal IPV (*B* = −.013, *SE* = .004, *p* < .001) and age 4 IPV (*B* = −.015, *SE* = .005, *p* = .001) were significantly associated with lower sensitivity. Prenatal income and age 4 child internalizing and externalizing symptoms were not significant predictors.

#### 3.3.2 Intrusive parenting

The overall model was not significant for intrusiveness, *χ^2^* (7) = 13.64, *p* = .058. Therefore, no further interpretation of group or covariate effects is presented.

#### 3.3.3 Unresponsive parenting

The overall model was significant for unresponsiveness, *χ^2^* (7) = 36.37, *p* = .001. However, postnatal maternal representation type did not significantly predict unresponsive parenting, Wald *χ* ^2^(2) = 5.13, *p* = .077. Regarding covariates, higher levels of prenatal IPV (*B* = .014, *SE* = .005, *p *= .003) and age 4 IPV (*B* = .022, *SE* = .010, *p *= .033) were both significantly associated with greater unresponsiveness. In addition, higher levels of child internalizing symptoms at age 4 significantly predicted greater unresponsive parenting (*B* = .069, *SE *= .031, *p* = .023). Prenatal income and child externalizing symptoms were not significant predictors.

### 3.4 Stability and change in working models related to parenting behaviors

#### 3.4.1 Remained balanced vs. became balanced

For the comparison between mothers who consistently maintained balanced representations from pregnancy to 1 year postpartum and mothers who shifted to balanced representations, the overall models for all parenting variables were not significant, *χ^2^* (6) = 5.39, *p* = .495; therefore, no further group or covariate effects were interpreted.

#### 3.4.2 Remained non‐balanced vs. became non‐balanced

For the comparison between mothers who consistently remained non‐balanced at both time points and those who shifted from balanced to non‐balanced, the overall model was significant for sensitivity, *χ^2^* (6) = 19.95, *p* = .003. Group differences were significant, Wald *χ* ^2^(1) = 7.35, *p* = .007. As presented in Table 4, pairwise comparisons revealed that mothers who were initially balanced and later became non‐balanced exhibited significantly higher sensitivity (*M* = 3.46, *SE* = 0.13) compared to those who remained non‐balanced (*M* = 3.00, *SE* = 0.09, *p* = .007).

**TABLE 4:** Changes in working models on parenting behaviors at 4 years postpartum.

Dependent variable	Comparison	Mean difference	*S.E*.	*χ^2^*	*p*	95% CI	
						*LL*	*UL*
Sensitivity	Balanced to balanced vs. non‐balanced to balanced	0.02	0.12	0.28	.867	−0.22	0.26
	Non‐balanced to non‐balanced vs. balanced to non‐balanced	−0.45^**^	0.17	7.35	.007	−0.78	−0.13
Intrusiveness	Balanced to balanced vs. non‐balanced to balanced	0.08	0.14	0.33	.564	−0.19	0.36
	Non‐balanced to non‐balanced vs. balanced to non‐balanced	0.27	0.27	1.00	.316	−0.25	0.79
Unresponsiveness	Balanced to balanced vs. non‐balanced to balanced	−0.12	0.16	0.55	.458	−0.43	0.20
	Non‐balanced to non‐balanced vs. balanced to non‐balanced	0.35^†^	0.18	3.76	.053	−0.00	0.71

Abbreviations: CI, confidence interval; LL, lower limit; UL, upper limit.

^†^ *p *< .10, ^*^ *p* < .05, and ^**^ *p* < .01;

For unresponsiveness, the overall model was significant, *χ* ^2^(6) = 15.48, *p* = .017; however, the group difference did not reach statistical significance, Wald *χ* ^2^(1) = 3.76, *p* = .053. The model for intrusive parenting was not significant, *χ* ^2^(6) = 5.39, *p* = .495.

Regarding covariates, prenatal IPV was significantly associated with sensitivity (*B* = −.012, *SE* = .005, *p* = .007), indicating that greater IPV exposure during pregnancy was related to lower maternal sensitivity in this transition model. Other covariates, including prenatal household income, age 4 IPV, and child internalizing and externalizing symptoms at age 4, were not significant predictors in these transition models.

## 4 DISCUSSION

This study examined associations between mothers’ prenatal and postnatal representations of their children and parenting behavior at age 4. A particularly notable finding was the enduring influence of prenatal representations on later caregiving, as balanced prenatal representations were associated with greater sensitivity and lower unresponsiveness. Postnatal representations showed more limited, domain‐specific associations, such that they were associated with sensitive parenting at age 4 but were not significantly related to intrusive or unresponsive behaviors. Furthermore, we found that even mothers who shifted from a balanced to a non‐balanced representation postnatally displayed more sensitive parenting than those who consistently remained non‐balanced. Together, these findings suggest that prenatal maternal representations, particularly balanced and disengaged representations, may establish a foundational framework that continues to guide caregiving behaviors beyond infancy.

In general, the hypothesized relationships between prenatal maternal representations and parenting at age 4 were confirmed, such that mothers with balanced prenatal representations displayed higher levels of sensitive parenting and lower levels of unresponsive parenting compared to mothers with disengaged representations. These results align with previous research highlighting the prenatal period as critical for forming maternal working models that guide later caregiving (Benoit et al., 1997; Dayton et al., 2010; Huth‐Bocks, Levendosky, Bogat, et al., 2004; Theran et al., 2005), and with the conclusions of a recent meta‐analytic review by Foley and Hughes (2018), which demonstrated that prenatal parental representations are robust predictors of later parent‐infant interaction quality. Importantly, our study extends these findings by showing that the effects of prenatal representations persist beyond infancy. In examining covariates within these models, higher levels of prenatal IPV and IPV at age 4 were associated with lower sensitivity and higher unresponsiveness, whereas household income and child internalizing and externalizing symptoms were not significant predictors.

These findings may be understood in light of the defining characteristics of balanced and disengaged representations. Mothers with balanced representations are characterized by emotional warmth and acceptance toward the child, as well as a coherent understanding of the child as a separate individual with their own thoughts, feelings, needs, and motivations (Zeanah et al., 1996). Such representations may facilitate a more flexible and accurate understanding of the child, enabling mothers to respond contingently and sensitively to their child's cues. These qualities may contribute to the stability of sensitive caregiving across developmental periods, even as children's needs become increasingly complex during the preschool years. In contrast, disengaged representations are characterized by emotional distance, indifference, and limited psychological investment in the child (Zeanah et al., 1996), which may reduce a mother's ability or motivation to attend to and respond appropriately to the child's needs.

Interestingly, although prior work with this sample found that prenatal distorted representations predicted intrusive parenting at age 1 (Dayton et al., 2010), our study found no such association at age 4. Mothers with prenatal distorted representations did not differ significantly from those with prenatal balanced or disengaged representations on any parenting outcomes. One possible explanation is the complex and multifaceted nature of distorted representations, which encompass various emotional and cognitive patterns. For example, some mothers with distorted representations may show intrusive behaviors driven by anxiety or confusion about their caregiving role, including hostile‐intrusive or frightening reactions, while others who are self‐focused or emotionally overwhelmed may display frightened or withdrawn, rather than intrusive, parenting behaviors (Schechter et al., 2008). Overall, the heterogeneous nature of distorted representations may help explain their weaker association with parenting behaviors observed at age 4.

Partially consistent with our hypotheses, postnatal representations predicted sensitive parenting at age 4, with balanced representations linked to greater sensitivity than disengaged ones. However, they were not significantly associated with intrusive or unresponsive parenting. These findings extend, but also qualify, previous studies linking postnatal representations to caregiving quality (Edwards et al., 2021; Korja et al., 2010; Sokolowski et al., 2007; Vreeswijk et al., 2012). Notably, the systematic review by Vreeswijk et al. (2012) found that balanced maternal representations assessed postnatally are consistently associated with sensitive, responsive caregiving, while non‐balanced representations predict less optimal parenting across a range of studies. However, most of this prior work has focused on younger children, from infancy to early toddlerhood, whereas parenting in our study was measured when children were 4 years old—a developmental period characterized by increased autonomy, emotional complexity, and social‐cognitive development (Alwaely et al., 2021; Erikson, 1963; Gomes & Pereira, 2014). As children grow more independent, mothers may continue to adapt their internal representations to meet evolving developmental demands. This ongoing adaptation may weaken the predictive utility of postnatal representations, whereas prenatal representations—formed before these complexities emerge—may exert a more enduring and clearer influence on parenting. When considering covariates in the postnatal representation models, both prenatal IPV and IPV at age 4 significantly predicted lower sensitivity and higher unresponsiveness, whereas income was not significant. Additionally, higher child internalizing symptoms at age 4 were associated with greater unresponsive parenting, consistent with well‐documented evidence that concurrent child characteristics contribute to variations in caregiving behavior during early childhood (Ahmad et al., 2025; Ciciolla et al., 2014; Serbin et al., 2015; Thompson et al., 2025).

The second aim of the study was to determine whether maintaining or transitioning between balanced and non‐balanced representations (from pregnancy to child age 1) affected parenting at age 4. Consistent with our hypothesis, mothers who shifted from balanced to non‐balanced representations demonstrated greater sensitivity compared to those who remained non‐balanced. Although a similar pattern emerged for unresponsiveness, this difference did not reach statistical significance. This suggests that earlier balanced representations may have a lasting influence on caregiving, even after a postnatal shift to non‐balanced representations, supporting prior theoretical and empirical work on the buffering role of early working models (Bowlby, 1988; Theran et al., 2005).

Interestingly, no significant differences emerged between mothers who remained balanced across both time points and those who shifted to balanced representations. One possible explanation is that both groups experienced a balanced representation at some point, which may have fostered sensitive caregiving. For mothers who shifted to balanced representations, this postnatal change may have mitigated the effects of earlier non‐balanced representations, leading to similar parenting outcomes as those who were consistently balanced. In contrast, those who remained non‐balanced did not benefit from such protective effects, resulting in less adaptive parenting. This pattern suggests that having a balanced representation at any stage—prenatal or postnatal—can positively influence parenting behaviors, though early representations may have a uniquely enduring effect, based on the other findings.

Notably, while non‐balanced prenatal representations may reflect emotional distance, confusion, or unrealistic perceptions of the child, they do not necessarily determine long‐term parenting behaviors when a positive shift occurs postpartum. Instead, they may be more malleable and open to change as mothers gain real‐life caregiving experience. The comparable parenting behaviors observed between mothers who shifted to balanced representations postnatally and those who were consistently balanced suggest that such postnatal improvements can help overcome the influence of earlier non‐balanced representations, fostering sensitive and adaptive caregiving. These findings underscore the importance of supporting such representational shifts, particularly for mothers who begin with non‐balanced representations.

Furthermore, in the stability and change analyses, prenatal IPV remained a significant predictor of lower sensitivity and higher unresponsiveness, while IPV at age 4 was not significant. Taken together with the absence of consistent income effects across all models, these findings suggest that while financial hardship may contribute to parenting stress, the emotional impact of IPV may exert a more robust and direct effect on caregiving quality in early childhood, emphasizing the need to consider relational trauma as a key contextual factor influencing parenting outcomes.

### 4.1 Limitations and strengths

The present study has several limitations. First, the small sample size within specific transition groups was small, which prevented a direct comparison between mothers who shifted from balanced to non‐balanced representations and those who shifted from non‐balanced to balanced. Second, the distribution of maternal representation classifications was uneven, with balanced representations being substantially more common than distorted representations. Especially, distorted representations were the least frequent classification during the prenatal period, resulting in relatively small subgroup sizes and potentially reducing statistical power to detect significant group differences. Consequently, the absence of significant associations involving distorted representations should be interpreted cautiously. This limitation may partially explain why distorted representations were not significantly associated with later parenting behaviors in the present study, despite previous findings linking distorted representations to hostile caregiving during infancy (Dayton et al., 2010). Future studies with larger samples are needed to more robustly examine how the timing and direction of representational shifts influence parenting behaviors and to clarify the associations between distorted and disengaged representations and later caregiving outcomes.

Third, maternal representations were not reassessed at age 4. This limits our ability to examine the influence of the most proximal representations on concurrent parenting behaviors, as representations may shift in response to the child's developmental changes and evolving parent–child interactions. Including a later assessment would provide a clearer picture of how maternal representations transition across the developmental period and offer additional insight into whether observed parenting behaviors are linked more closely to earlier representations or to more recent perceptions of the child.

Fourth, although child characteristics such as temperament, health status, and developmental functioning likely reciprocally influence mother–child interactions and parenting behaviors, the present study only included child behavioral problems at age 4 as covariates. Broader dimensions of child functioning were not assessed. Future research should incorporate more comprehensive measures of child characteristics to better isolate the unique contribution of maternal representations to parenting behaviors. Finally, the present study was limited by reduced statistical power due to missing parenting outcome data at age 4. Because complete‐case analyses were used, reduced power may have limited our ability to detect smaller effects. Additionally, missingness in parenting outcomes may have been related to caregiving behaviors themselves, and thus the findings should be interpreted with this limitation in mind. Future research is needed to replicate these results using methods that more fully address outcome missingness.

Despite these limitations, this study also has several notable strengths. First, the study included a diverse sample in terms of both racial/ethnic background and socioeconomic risk factors. This diversity enhances the generalizability of the findings across various populations. Furthermore, the study included participants who faced significant disadvantages, such as IPV exposure during pregnancy and low household income. By focusing on this vulnerable population, the study provides valuable insights into how maternal representations and parenting behaviors are shaped under challenging circumstances, with important implications for interventions targeting at‐risk families.

Second, the longitudinal prospective design allowed us to assess maternal representations during pregnancy and predict parenting behaviors when the child was 4 years old, strengthening the causal inference between maternal representations and parenting behaviors. To our knowledge, this is the first study to examine the relationship between both prenatal and postnatal maternal representations and parenting behaviors during early childhood. By integrating representations across time, our findings extend previous research primarily focused on the enduring impact of prenatal representations on parenting measured during infancy. Importantly, our results confirm that the influence of prenatal representations lasts until the child is 4 years old, highlighting the long‐term significance of early maternal mental models. This broader developmental window offers a more comprehensive understanding of how maternal representations shape parenting behaviors over time. Moreover, we employed multiple methods in this study, including structured interviews and observational assessments of parenting behaviors. The use of mother‐child interaction observations, rather than relying solely on self‐reported measures, allowed us to capture real‐time parenting behaviors more accurately, thus increasing the validity of our assessments.

### 4.2 Implications for practice

Given the critical role of early parenting in child development and our finding that balanced representations at any time point are beneficial, the study underscores the importance of identifying and monitoring maternal representations during pregnancy or the early postnatal period. Furthermore, the findings suggest that maternal representations are not static and may shift over time, highlighting the need to support mothers—particularly those who maintain non‐balanced representations across both periods—through interventions that promote reflective awareness of their internal working models.

For example, Child–Parent Psychotherapy (CPP) and its prenatal adaptation, Perinatal Child–Parent Psychotherapy (P‐CPP), are evidence‐based, trauma‐informed interventions designed to enhance the caregiver‐child relationship, particularly in families exposed to IPV (Lieberman et al., 2018, 2011). These interventions aim to restore safety and trust, foster emotional regulation, and support resuming child age‐appropriate developmental milestones by increasing the caregiver's reflective capacity regarding its critical impact on child development. Our findings may help refine and target these interventions by identifying which mothers, especially those with persistently non‐balanced representations, are most likely to benefit from such support. In particular, the results highlight the importance of early screening and engagement during pregnancy, thereby supporting the value of implementing P‐CPP in the perinatal period and expanding access to such interventions for high‐risk and under‐resourced families.

Additionally, understanding the broader contextual factors that influence maternal representations—such as stress, IPV exposure, and socioeconomic adversity—is essential for informing both prevention and intervention efforts. Finally, the findings emphasize the importance of ongoing support for mothers, particularly those facing chronic stressors such as IPV or financial strain, as these factors may contribute to shifts in maternal representations. While further research is needed to explore the mechanisms underlying these shifts, the results suggest that maternal representations remain an important area of study in understanding long‐term parenting outcomes.

## CONFLICT OF INTEREST STATEMENT

The authors declare no conflicts of interest.

## Data Availability

The data that support the findings of this study are available from the corresponding author upon reasonable request.
